# Serum Metabonomics Reveals Risk Factors in Different Periods of Cerebral Infarction in Humans

**DOI:** 10.3389/fmolb.2021.784288

**Published:** 2022-02-15

**Authors:** Guoyou Chen, Li Guo, Xinjie Zhao, Yachao Ren, Hongyang Chen, Jincheng Liu, Jiaqi Jiang, Peijia Liu, Xiaoying Liu, Bo Hu, Na Wang, Haisheng Peng, Guowang Xu, Haiquan Tao

**Affiliations:** ^1^ College of Pharmacy, Harbin Medical University-Daqing, Daqing, China; ^2^ Department of Anesthesia, Zhuhai Hospital Affiliated with Jinan University, Zhuhai, China; ^3^ Key Laboratory of Separation Science for Analytical Chemistry, Dalian Institute of Chemical Physics, Chinese Academy of Sciences, Dalian, China; ^4^ Academic Affairs Office, Harbin Medical University-Daqing, Daqing, China; ^5^ Department of Clinical Laboratory, Second Affiliated Hospital of Harbin Medical University, Harbin, China; ^6^ Department of Neurosurgery, Second Affiliated Hospital of Harbin Medical University, Harbin, China; ^7^ Cerebrovascular Diseases Department, Zhuhai Hospital Affiliated with Jinan University, Zhuhai, China

**Keywords:** cerebral infarction, different periods, human serum, metabonomics, key metabolites and pathways

## Abstract

Studies of key metabolite variations and their biological mechanisms in cerebral infarction (CI) have increased our understanding of the pathophysiology of the disease. However, how metabolite variations in different periods of CI influence these biological processes and whether key metabolites from different periods may better predict disease progression are still unknown. We performed a systematic investigation using the metabonomics method. Various metabolites in different pathways were investigated by serum metabolic profiling of 143 patients diagnosed with CI and 59 healthy controls. Phe-Phe, carnitine C18:1, palmitic acid, cis-8,11,14-eicosatrienoic acid, palmitoleic acid, 1-linoleoyl-rac-glycerol, MAG 18:1, MAG 20:3, phosphoric acid, 5α-dihydrotestosterone, Ca, K, and GGT were the major components in the early period of CI. GCDCA, glycocholate, PC 36:5, LPC 18:2, and PA showed obvious changes in the intermediate time. In contrast, trans-vaccenic acid, linolenic acid, linoleic acid, all-cis-4,7,10,13,16-docosapentaenoic acid, arachidonic acid, DHA, FFA 18:1, FFA 18:2, FFA 18:3, FFA 20:4, FFA 22:6, PC 34:1, PC 36:3, PC 38:4, ALP, and Crea displayed changes in the later time. More importantly, we found that phenylalanine metabolism, medium-chain acylcarnitines, long-chain acylcarnitines, choline, DHEA, LPC 18:0, LPC 18:1, FFA 18:0, FFA 22:4, TG, ALB, IDBIL, and DBIL played vital roles in the development of different periods of CI. Increased phenylacetyl-L-glutamine was detected and may be a biomarker for CI. It was of great significance that we identified key metabolic pathways and risk metabolites in different periods of CI different from those previously reported. Specific data are detailed in the Conclusion section. In addition, we also explored metabolite differences of CI patients complicated with high blood glucose compared with healthy controls. Further work in this area may inform personalized treatment approaches in clinical practice for CI by experimentally elucidating the pathophysiological mechanisms.

## Introduction

Cerebral infarction (CI) occurs suddenly, usually with more severe sequelae (Tarhouni-Jabberi et al., 2017), and leads to a high rate of disability and mortality in cases of untimely treatment (Klingseisen and Jackson, 2011). It is very necessary to find the potential biomarkers and elucidate the mechanisms of CI. Possible protein markers have been identified (Klingseisen and Jackson, 2011), and studies have also shown that linoleic acid and amino acids are related to the risk of adverse cardiovascular events (Marklund et al., 2019; Sidorov et al., 2020). Gut microbiota can regulate brain disorders, suggesting that CI might also involve the microbiota (Zhu et al., 2020). In addition, magnetic resonance spectroscopy can now be used to monitor cerebral infarction events in clinical settings (Hyacinthe et al., 2020). With an increasing number of people developing CI, the study and identification of key metabolites for rapid diagnosis and understanding physiological and pathological mechanisms are increasingly important.

Metabonomics is a potent method in disease phenotype studies and plays an important role in a number of aspects such as biomarker discovery, the origin and development of a disease, and its personalized treatment (Nicholson et al., 2005; Huang et al., 2013; Wishart, 2016). Although some metabonomics studies of acute ischemic stroke exist (Liu et al., 2017; Au, 2018; Ke et al., 2019), no studies have looked at the different stages of CI, and indicative metabolites and definite causes of CI at different stages are not fully understood. Thus, our study is the first to look at the different stages of CI in relation to metabolites. We performed serum metabolic profiling of 143 cerebral infarction patients and 59 healthy controls using ultra performance liquid chromatography–triple time of flight mass spectrometry (UPLC-Triple-TOF-MS). We explored more than 100 metabolites from four different periods of cerebral infarction and a diabetic group in human serum, all of which were closely correlated with amino acids (AA), bile acids (BA), stearates, carnitines, sphingolipids, phosphatidylcholines (PC), lysophosphatidyl cholines (LPC), free fatty acids (FFA), sterols, and other metabolites. In addition, over 40 metabolites were found in the cerebral infarction and diabetes (T) groups compared with those in healthy controls (N), and over 30 metabolites from one or more periods were discovered between the cerebral infarction (A–D) and healthy controls (N). Specifically, the first batch, as the discovery group: healthy controls, n = 41; CI occurred within 3 days was defined as group A (31 CIs), 3–5 days was defined as group B (17 CIs), 5–7 days of CI was defined as group C (13 CIs), after 7 days of CI was defined as group D (19 CIs), and CI patients with glycosuria were defined as group T (20 CIs). The data from the second group were used to validate the results obtained from the first group: healthy controls, *n* = 18; group A (7 CIs), group B (8 CIs), group C (5 CIs), group D (11 CIs), and group T (12 CIs). These metabolites regulate various pathways including amino acid metabolism, tricarboxylic acid cycle, bile acid metabolism, and multiple lipid pathways in the human body. These results explore new potential therapeutic target metabolites and provide important reference in judging the timing and development stages of brain infarction based on metabonomic analysis. Otherwise, clinical recurrence of different stages of cerebral infarction is only by instrumentation such as CT and NMR. The key metabolites we discovered will be beneficial for rapid clinical diagnosis and individualized treatment of patients at different stages of cerebral infarction.

## Materials and Methods

### Sample Collection of Varying Stages of CI

All patients were recruited from the Second Affiliated Hospital of the Harbin Medical University. This study was approved by the Ethics Committee of the Harbin Medical University (HMUDQ2019082601). Cerebral infarction was diagnosed in 2016 according to the Chinese guidelines for cerebrovascular disease prevention and treatment. The inclusion and exclusion criteria are as follows: 1) sudden onset; 2) focal nerve defects (weakness or numbness on one face or limb, language disorders, etc.), a few are comprehensive neurological defects;3) the duration of symptoms or signs has no limit when imaging shows clear ischemic lesions or has continued for more than 24 h when imaging indicated lesions are lacking; (4) excluded the non-vascular etiology; and 5) Brain CT/MRI excluded cerebral hemorrhage. According to the time from onset, patients within 3 days to 7 days away were randomly divided into five groups. Specifically, time within 3 days of cerebral infarction was defined as group A (31 CIs), time after 3 days but within 5 days of cerebral infarction was defined as group B (17 CIs), time after 5 days but within 7 days of cerebral infarction was defined as group C (13 CIs), time after 7 days of cerebral infarction was defined as group D (19 CIs), and cerebral infarction patients with glycosuria were defined as group T (20 CIs). According to the Second Affiliated Hospital’s routine clinical laboratory procedures of the Harbin Medical University, serum was kept in cryotubes and stored at −80°C in freezers after biochemical tests. The first batch of serum (test group) for non-targeted metabolomic analysis was collected from 100 CI patients and 41 healthy controls from November 2016 to January 2017. The second batch of serum (validation group) was collected from 48 CI patients and 19 healthy controls for validation analysis from January to February 2017. Two batches of serum were needed because of the limited blood collected from each person and the expansion of the population size. There was no significant difference in age and gender distribution across groups. The sample information is summarized in Table 1 and Supplementary Table S1.

**TABLE 1:** Patient information.

First batch	Healthy controls	A group	B group	C group	D group	T group
Number (n)	41	31	17	13	19	20
Gender	13 males	10 males	5 males	7 males	10 males	13 males
	28 females	8 females	2 females	2 females	7 females	6 females
	0 missing	13 missing	10 missing	4 missing	2 missing	1 missing
Age (years)	60.4 ± 4.6	60.4 ± 9.3	62.6 ± 11.0	62.9 ± 3.8	62.2 ± 8.1	60.8 ± 4.5
UA (IU/mL)	292.2 ± 64.3	314.4 ± 96.8	318.7 ± 45.7	274.4 ± 76.5	277.5 ± 53.5	324.9 ± 100.1
GLU	5.04 ± 0.5	9.3 ± 5.2*	5.4 ± 0.2^ab^	6.6 ± 2.4	6.7 ± 2.1	12.5 ± 5.1* ^,^ ^bt^ ^,^ ^ct^ ^,^ ^dt^
T_CH	4.7 ± 0.7	4.9 ± 1.4	5.4 ± 1.1	5.1 ± 1.1	5.1 ± 1.2	4.4 ± 1.2
TG	1.0 ± 0.5	2.8 ± 2.1*	2.0 ± 0.9*	1.7 ± 0.6*	1.5 ± 0.6*	2.0 ± 0.8*
HDL_C	1.4 ± 0.3	1.5 ± 1.0	1.1 ± 0.3	1.5 ± 0.7	1.3 ± 0.5	1.1 ± 0.2*
LDL_C	2.6 ± 0.6	2.7 ± 0.9	3.5 ± 0.7*	2.8 ± 0.8	3.2 ± 1.0	2.8 ± 1.1
Second batch	Healthy controls	A group	B group	C group	D group	T group
Number (n)	18	7	8	5	11	12
Gender	10 males	5 males	5 males	2 males	5 males	7 males
	8 females	2 females	3 females	3 females	6 females	5 females
Age (years)	60.6 ± 3.8	60.7 ± 5.9	60.9 ± 4.6	61.8 ± 2.3	61.7 ± 3.7	60.9 ± 5.3
UA (IU/mL)	5.2 ± 0.3	326.2 ± 104.3	305.3 ± 83.6	272.8 ± 66.8	300.8 ± 81.7	319.4 ± 113.0
GLU	4.5 ± 0.7	5.7 ± 1.0	5.3 ± 0.3	5.2 ± 0.4	6.9 ± 1.8	9.7 ± 4.3*
T_CH	1.1 ± 0.4	5.3 ± 1.4	4.4 ± 0.8	3.9 ± 0.7	4.1 ± 1.3	4.9 ± 1.2
TG	1.5 ± 0.4	2.9 ± 3.7	1.8 ± 1.1	1.7 ± 0.7	2.7 ± 1.2*	1.7 ± 0.9
HDL_C	2.4 ± 0.5	1.2 ± 0.2	1.3 ± 0.4	1.2 ± 0.3	1.2 ± 0.3	1.2 ± 0.5
LDL_C	2.4 ± 0.5	2.8 ± 1.0	2.6 ± 0.6	2.2 ± 0.4	2.4 ± 1.1	3.1 ± 0.8*

Note: N, healthy controls; n first = 41, n second = 18; A, time within 3 days of cerebral infarction, n first = 31, n second = 7; B, time after 3 days but within 5 days of cerebral infarction, n first = 17, n second = 8; C, time after 3 days but within 5 days of cerebral infarction, n first = 13, n second = 5; D, time after 7 days of cerebral infarction, n first = 19, n second = 11; T, cerebral infarction patients with glycosuria, n first = 20, n second = 12.

* *p* < 0.05, A,B,C, and D group compared with N group.

ab *p* < 0.05 A group compared with B group;

bt *p* < 0.05 B group compared with T group;

ct *p* < 0.05 C group compared with T group;

dt *p* < 0.05 D group compared with T group; Mean ± SD, two-tailed Mann–Whitney *U* test.

### Non-Targeted Metabonomic Analysis of Various Phases of CI

We extracted metabolites from 200 µl of serum with four volumes of acetonitrile containing 12 internal standards (carnitine C 2:0-d3, carnitine C 16:0-d3, lysophosphatidylcholine (LPC) 12:0, LPC 19:0, tryptophan-d5, FFA 16:0-d3, FFA 18:0-d3, chenodeoxycholic acid-d4, tryptophan-d5, triglyceride (TG) 45:0, sphingomyelin (SM) 12:0/30:1, and phosphatidylethanolamine (PE) 30:0) (Zhao et al., 2014). Internal standards were added to each sample in order to check data from previous reports. After 15 min of centrifugation at 13,000 g, the supernatant was distributed into two aliquots and dried using a vacuum centrifuge at 4°C before the positive ion and negative ion mode tests. Aliquots were reconstituted in 100 μl of acetonitrile/water (2:8) and analyzed using a Waters ACQUITY UPLC (Waters Corp, Milford, United States), coupled with an AB SCIEX TripleTOF 5,600 System (ABSCIEX, Framingham, United States) (Zhao et al., 2014). The injection volume was 6 μl for each run. Quality control (QC) samples were carried out after every six serum samples, which were obtained by mixing 10 μl from each sample.

We used a 2.1 × 100 mm ACQUITYTM 1.7 μm C8 BEH column (Waters, Ireland) as a stationary phase in positive ion mode. Both water (phase A) and acetonitrile (phase B), including 0.1% formic acid, were the mobile phases. The UPLC was subjected to gradient elution: 95% A for 1 min, changed linearly to 100% B within 24 min, and then held for 4 min. A 2.1 × 100 mm ACQUITYTM 1.8 μm T3 HSS column (Waters, Ireland) was used for LC separation in negative ion mode (Zhao et al., 2014). Both water (phase C) and 95% methanol/water (phase D), containing 6.5 mM NH_4_HCO_3_, were the mobile phases. The first gradient elution was 2% D for 1 min, which then changed linearly to 100% D within 18 min, and finally maintained for 4 min. The flow was 0.35 ml/min, and the column temperature was 50°C (Zhao et al., 2014).

Data were obtained in full scan mode from m/z 80 to 1,000 with a cycle time of 275 ms. The MS parameters were set as follows: ion spray voltage, 5500 V (positive ion) and 4500 V (negative ion); curtain gas, 35 PSI; ion source gas 1, 50 PSI; ion source gas 2, 50 PSI; and interface heater temperature, 500°C (Zhao et al., 2014; Hu et al., 2017; Chew et al., 2018; Gu et al., 2018).

### Data Collection and Analysis

A Markerview workstation (AB SCIEX, United States) was used to extract and align the non-targeted metabonomic data. Internal standards were selected based on the lowest relative standard deviation value in the QC sample. Before further data analysis, we calibrated the intensity of each peak by its suitable internal standard and integrated the positive and negative ion non-targeted data into one table. We utilized the SIMCA-P software (version 14.1; Umetrics, Umea, Sweden) for multivariate statistical analysis. After unit variance scaling, principal component analysis (PCA) and orthogonal partial least-squares discriminant analysis (OPLS-DA) were applied to distinguish between healthy controls and patients with CI at different stages (Zhao et al., 2014; Li et al., 2015; Fan et al., 2018).

To prevent model overfitting, we conducted a permutation test. For non-targeted metabonomics, we used metabolites with variable importance in the projection values (VIP) larger than one for analysis (Kuboniwa et al., 2016; Li et al., 2020). The Wilcoxon Mann–Whitney test was used to identify significantly different metabolites. Statistical significance was defined as *p*< 0.05, and *p*< 0.10 as a criterion for the false discovery rate found in multiple comparisons. We demonstrated the relationship between different metabolites using the MeV4.5.1 version of software (Saeed et al., 2003). In Figure 3 to Figure 9, all data are the mean ±SEM. *p<0.05 vs. N group; **p<0.01 vs. N group; bt, *p*<0.05 B group vs. T group; dt, *p*<0.05 D group vs. T group; two-tailed Mann–Whitney *U* test. Raw data were presented in Supplementary Table S2.

## Results

### Metabonomic Analysis in Various Stages of Cerebral Infarction Samples by UPLC-TOF-MS

**FIGURE 1:**
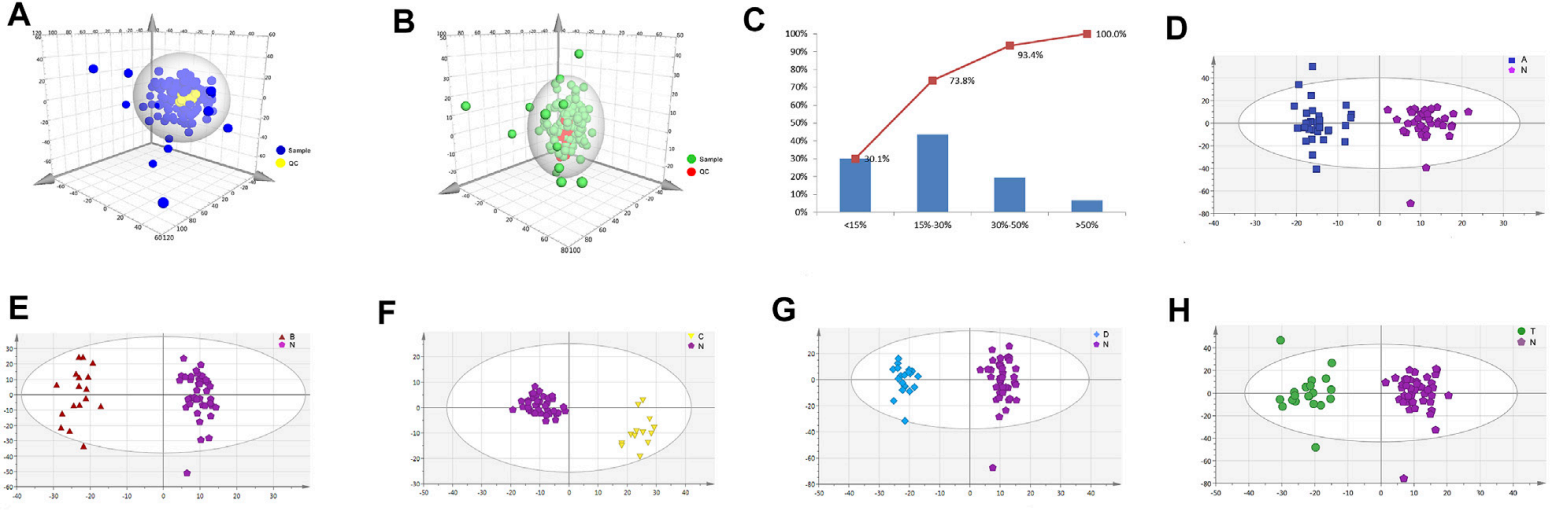
Metabolic profiling analysis of different periods of cerebral infarction in humans. **(A)** Score plot of the samples using PCA model in positive ions. **(B)** Score plot of the samples using PCA model in negative ions. **(C)** RSD (relative standard deviation) distribution of the ions in QC samples. **(D)** Score plot of samples from healthy controls (N) and attacks within 3 days of cerebral infarction (A) group using PLS-DA model. **(E)** Score plot of samples from healthy controls (N) and onsets after 3 days but within 5 days of cerebral infarction (B) group using PLS-DA model. **(F)** Score plot of samples from healthy controls (N) and seizures after 5 days but within 7 days of cerebral infarction (C) group using PLS-DA model. **(G)** Score plot of samples from healthy controls (N) and attacks after 7 days of cerebral infarction (D) group using PLS-DA model. **(H)** Score plot of samples from healthy controls (N) and cerebral infarction patients with glycosuria (T) group using PLS-DA model. N, healthy controls, *n* = 41; A, attacks within 3 days of cerebral infarction, *n* = 31; B, onsets after 3 days but within 5 days of cerebral infarction, *n* = 17; C, onsets after 3 days but within 5 days of cerebral infarction, *n* = 13; D, attacks after 7 days of cerebral infarction, *n* = 19; T, cerebral infarction patients with glycosuria group, *n* = 20.

To explore key metabolites from serum metabolism at different periods of cerebral infarction, serum metabolic profiles from the different groups (N, A, B, C, D, and T) were analyzed by UPLC-TOF-MS. QC data were also obtained to ensure dependability. High reproducibility of QC data indicated high quality results (Figures 1A,C). The relative standard deviation value of 73% (2301 ions) of the total ions (3140 ions) in the QC sample was less than 30% (Figure 1B). We performed OPLS-DA to further explore key metabolites from each group (Figures 1D–H). A clear differentiation was observed between groups A and N (Figure 1D), B and N (Figure 1E), C and N (Figure 1F), D and N (Figure 1G), and T and N (Figure 1H), respectively. All OPLS-DA models were reliable because none of the permutation tests had overfitting. The results obtained here were then used to explore critical metabolites and multiple metabolic pathways at different periods. VIP and *p* value data were presented in Supplementary Table S3.

### Metabolic Pathway Analysis in Different Periods of CI by the Online MetaboAnalyst Website

**FIGURE 2:**
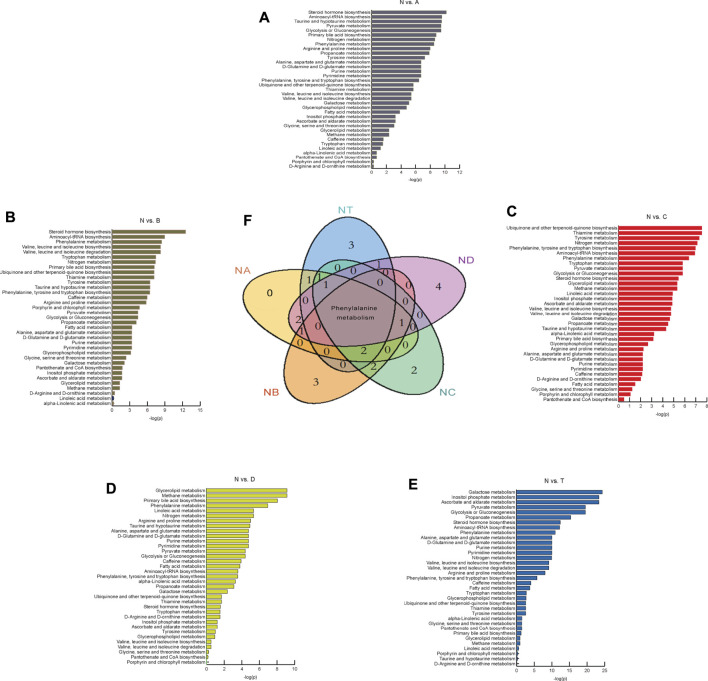
Differential metabolites found by metabolomics analysis and metabolic pathway analysis. **(A–E)** It represents the results of metabolic pathway in different treatment groups. The abscissa is $-\log_{\text{e}}\left(\text{p}\right)$ , the ordinate is metabolic pathway. **(F)** It presents the relationship of amino acid pathways in different treatment groups in the Venn diagram. Venn diagrams of samples from healthy controls (N) and cerebral infarction **(A–D)** group and cerebral infarction and glycosuria (T) group. N, healthy controls, *n* = 41; A, attacks within 3 days of cerebral infarction, *n* = 31; B, onsets after 3 days but within 5 days of cerebral infarction, *n* = 17; C, onsets after 3 days but within 5 days of cerebral infarction, *n* = 13; D, attacks after 7 days of cerebral infarction, *n* = 19; T, cerebral infarction patients with glycosuria group, *n* = 20.

We investigated metabolic pathways between the N group and various stages of the cerebral infarction group (Figures 2A–E). After comparison with healthy people, thirty-five metabolic pathways were identified and explored. Furthermore, we focused on the top ten important pathways that differed between the A, B, C, D, or T groups and N (Figure 2F). For instance, the top ten pathways that differed between the A and N groups were steroid hormone biosynthesis, aminoacyl-tRNA biosynthesis, taurine and hypotaurine metabolism, pyruvate metabolism, glycolysis or gluconeogenesis, primary bile acid biosynthesis, nitrogen metabolism, phenylalanine metabolism, arginine and proline metabolism, and propanoate metabolism. Importantly, our results revealed that phenylalanine metabolism was always involved in the development of cerebral infarction within 3 to 7 days. Interestingly, three pathways that differed only between the T and N groups were found. They were galactose metabolism, inositol phosphate metabolism, and ascorbate and aldarate metabolism. There were also only three pathways differentially active between the B and N groups. They are valine, leucine, and isoleucine biosynthesis (Gu et al., 2018); valine, leucine, and isoleucine degradation (Tong et al., 2020); and tryptophan metabolism, ubiquinone, and other terpenoid–quinone biosynthesis (Tong et al., 2020). Tyrosine metabolism and phenylalanine, tyrosine, and tryptophan biosynthesis differed significantly between the C and N groups. Finally, the four pathways differed between the D and N groups: glycerolipid metabolism, methane metabolism, linoleic acid metabolism, and d-glutamine and d-glutamate metabolism. These findings reveal that different pathways play different roles in the progression of CI over time, helping us to further explore the etiopathogenesis of different stages of cerebral infarction.

### Changes in Carbohydrates and Amino Acids at Different Times of Cerebral Infarction

**FIGURE 3:**
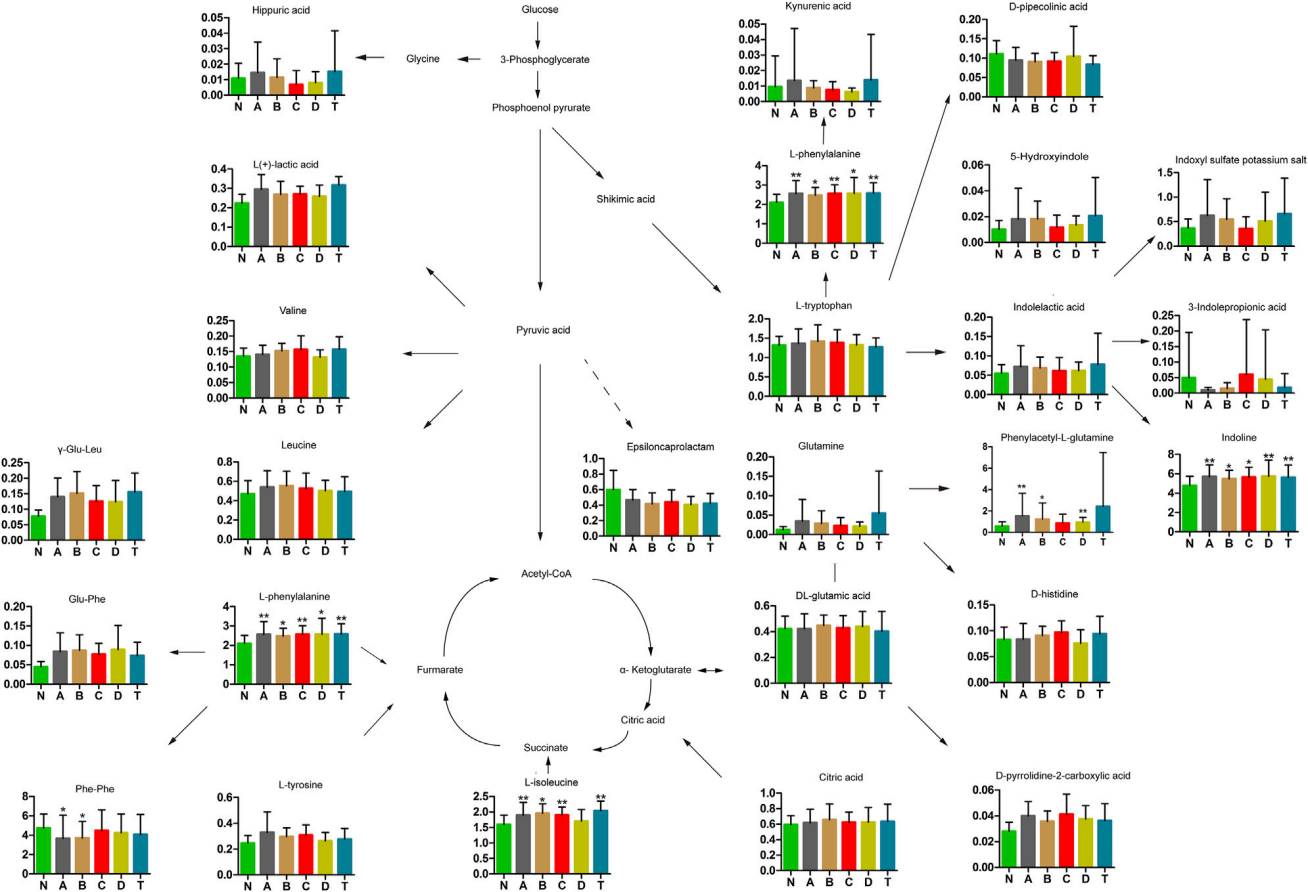
Changes in carbohydrates and amino acids of cerebral infarction. N, healthy controls, *n* = 41; A, attacks within 3 days of cerebral infarction, *n* = 31; B, onsets after 3 days but within 5 days of cerebral infarction, *n* = 17; C, onsets after 3 days but within 5 days of cerebral infarction, *n* = 13; D, attacks after 7 days of cerebral infarction, *n* = 19; T, cerebral infarction patients with glycosuria group, *n* = 20. All data are the mean ±SEM. *p < 0.05 vs. N group; **p < 0.01 vs. N group, two-tailed Mann–Whitney *U* test.

Previous reports found carbohydrate and amino acid metabolites in glycuresis, angiocardiopathy, and therioma (Tabas, 2010). Therefore, we further analyzed serum carbohydrate and amino acid metabolites at various periods of CI. As shown in Figure 3, results revealed that five types of metabolites were changed significantly compared to those in the N group. Specifically, four metabolites, L-phenylalanine, phenylacetyl-L-glutamine, indoline, and L-isoleucine (except for the D group), were dramatically elevated at different periods of cerebral infarction compared to those in the N group, while Phe-Phe was dramatically decreased in the A and B group. Finally, 20 types of metabolites were identified that did not dramatically change between the different periods of cerebral infarction and the N group.

### Related Metabolites in Bile Acids in Different Periods of CI

**FIGURE 4:**
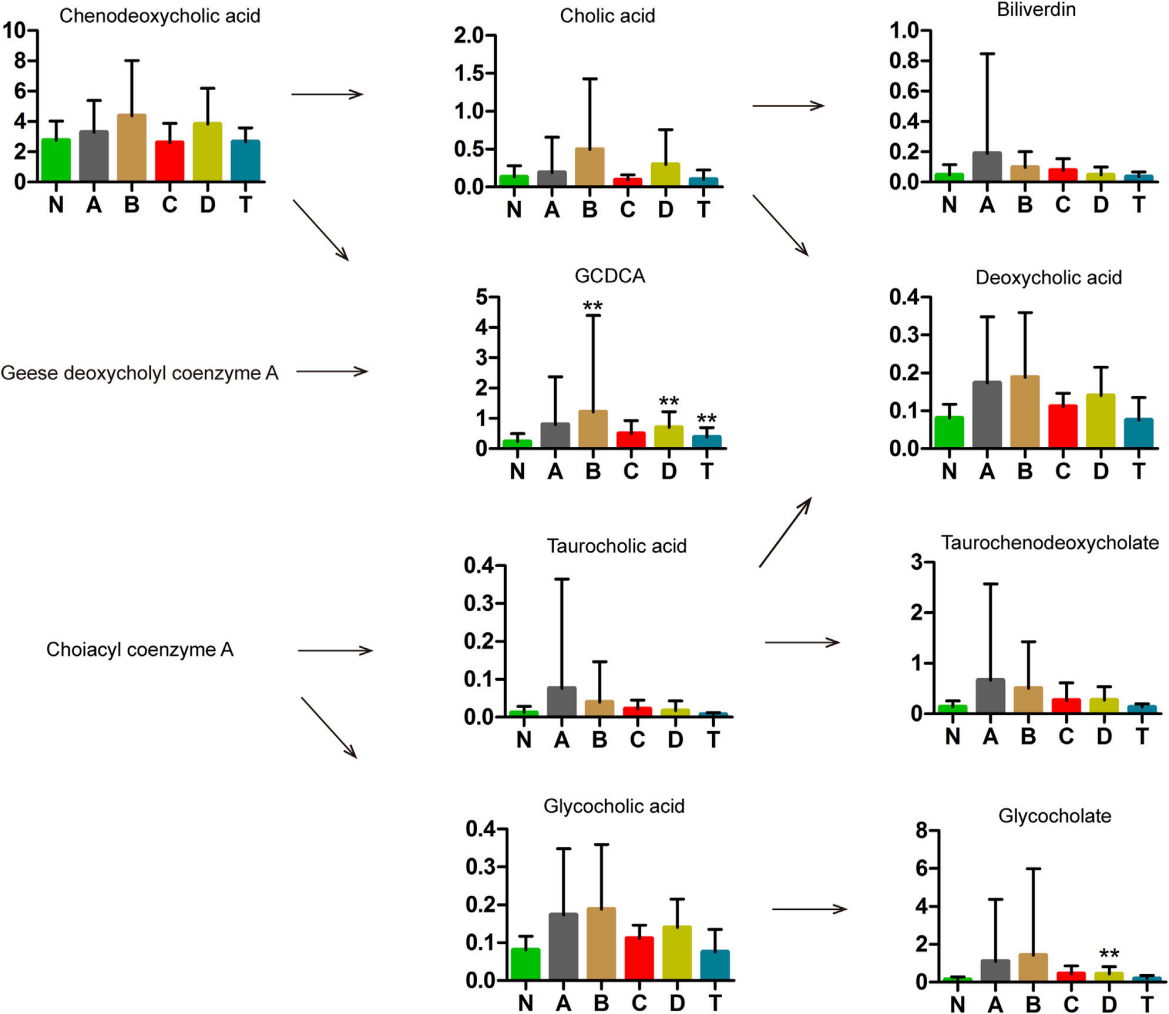
Metabolomics analysis in bile acids of cerebral infarction. N, healthy controls, n = 41; A, attacks within 3 days of cerebral infarction, *n* = 31; B, onsets after 3 days but within 5 days of cerebral infarction, *n* = 17; C, onsets after 3 days but within 5 days of cerebral infarction, *n* = 13; D, attacks after 7 days of cerebral infarction, *n* = 19; T, cerebral infarction patients with glycosuria group, *n* = 20. All data are the mean ±SEM. **p < 0.01 vs. N group, two-tailed Mann–Whitney *U* test.

Some components of bile acids have been reported to have protective effects against stroke and cerebral ischemia injury (Tabas, 2010; Wang et al., 2018). We detected nine types of bile acids in the sera of patients with CI (Figure 4). Specifically, glycochenodeoxycholate (GCDCA) showed a marked increase in B, D, and T groups compared with the N group, while glycocholate showed an obvious increase only between D and N groups. With the exception of GCDCA and glycocholate, all the other seven substances showed no change between the CI groups and N.

### Regulated Carnitines in Patients With Varying Stages of CI

**FIGURE 5:**
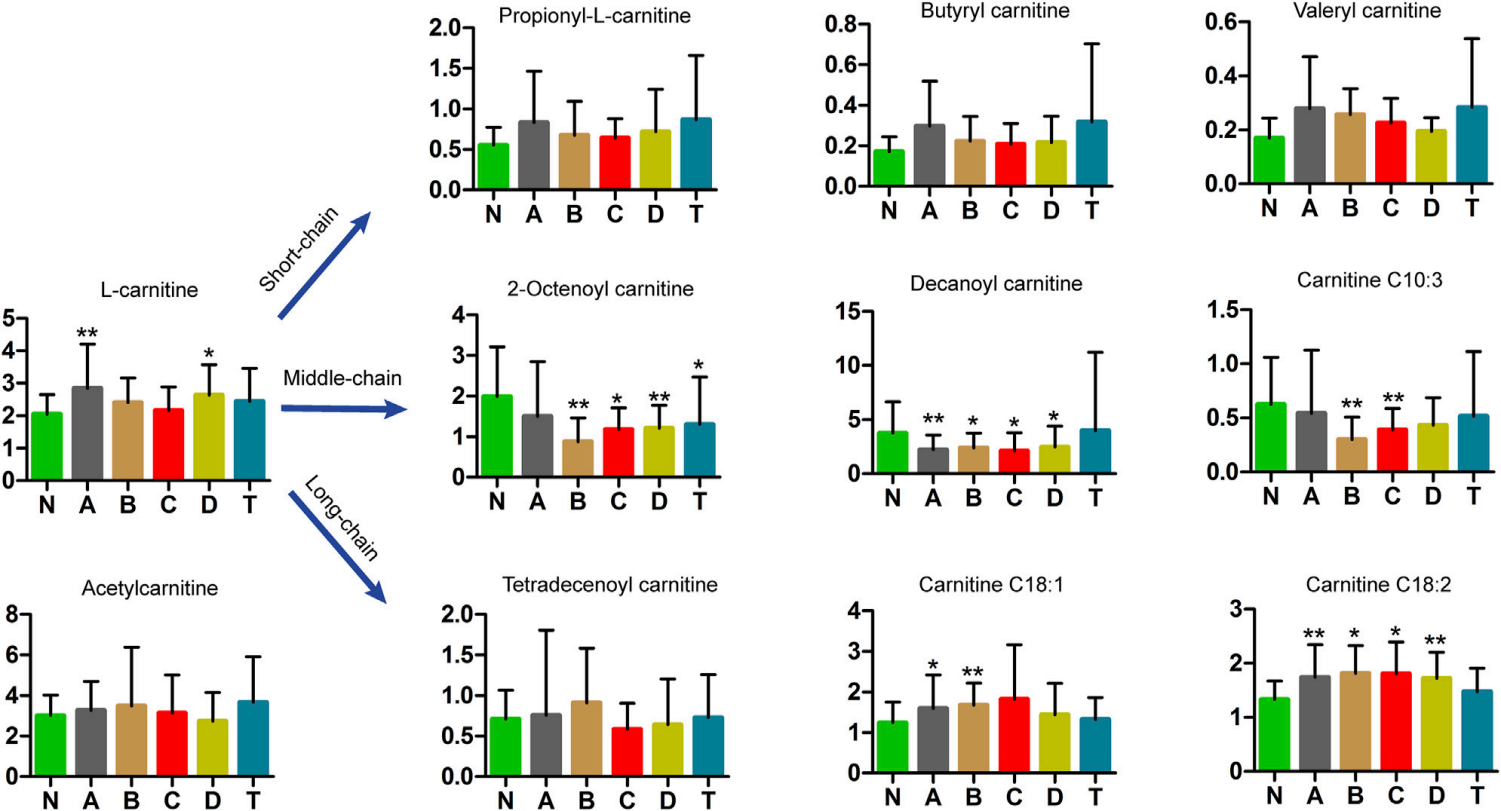
Metabolomics analysis in carnitines of cerebral infarction. N, healthy controls, *n* = 41; A, attacks within 3 days of cerebral infarction, *n* = 31; B, onsets after 3 days but within 5 days of cerebral infarction, *n* = 17; C, onsets after 3 days but within 5 days of cerebral infarction, *n* = 13; D, attacks after 7 days of cerebral infarction, *n* = 19; T, cerebral infarction patients with glycosuria group, *n* = 20. All data are the mean ±SEM. *p < 0.05 vs. N group; **p < 0.01 vs. N group, two-tailed Mann–Whitney *U* test.

Carnitine plays an important role in mitochondrial metabolism and in modulating the ratio of coenzyme A (CoA): acyl-CoA (Zhang et al., 2012a). Studies have shown that l-carnitine (LC) and acetyl-l-carnitine (ALC) were highly effective against ischemic injury in neuronal cells *in vitro*, but ALC also played a protective role in neurons after ischemic injury *in vivo*. Using UPLC-MS analysis, we detected free carnitine, acetyl carnitine, and nine carnitines with chain lengths from 3 to 18 carbon units (Figure 5). The most notable differences were observed for medium-chain carnitines, most of which were lower in CI serum than in the N group. Specifically, 2-octenoyl carnitine in B, C, D, and T groups was lower than in N; decanoyl carnitine in A, B, C, and D groups was lower than in N; and carnitine C10:3 in B and C groups was lower than in N. In contrast, long-chain carnitines were higher in CI groups than those in N. In particular, carnitine C18:1 in A and B groups is higher than in N; carnitine C18:2 in A, B, C, and D groups is higher than in N. However, short-chain acylcarnitines showed no differences. These results are highly interesting but need explanation.

### Stearate Metabolic Pathway Analysis in the Various Phases of CI

**FIGURE 6:**
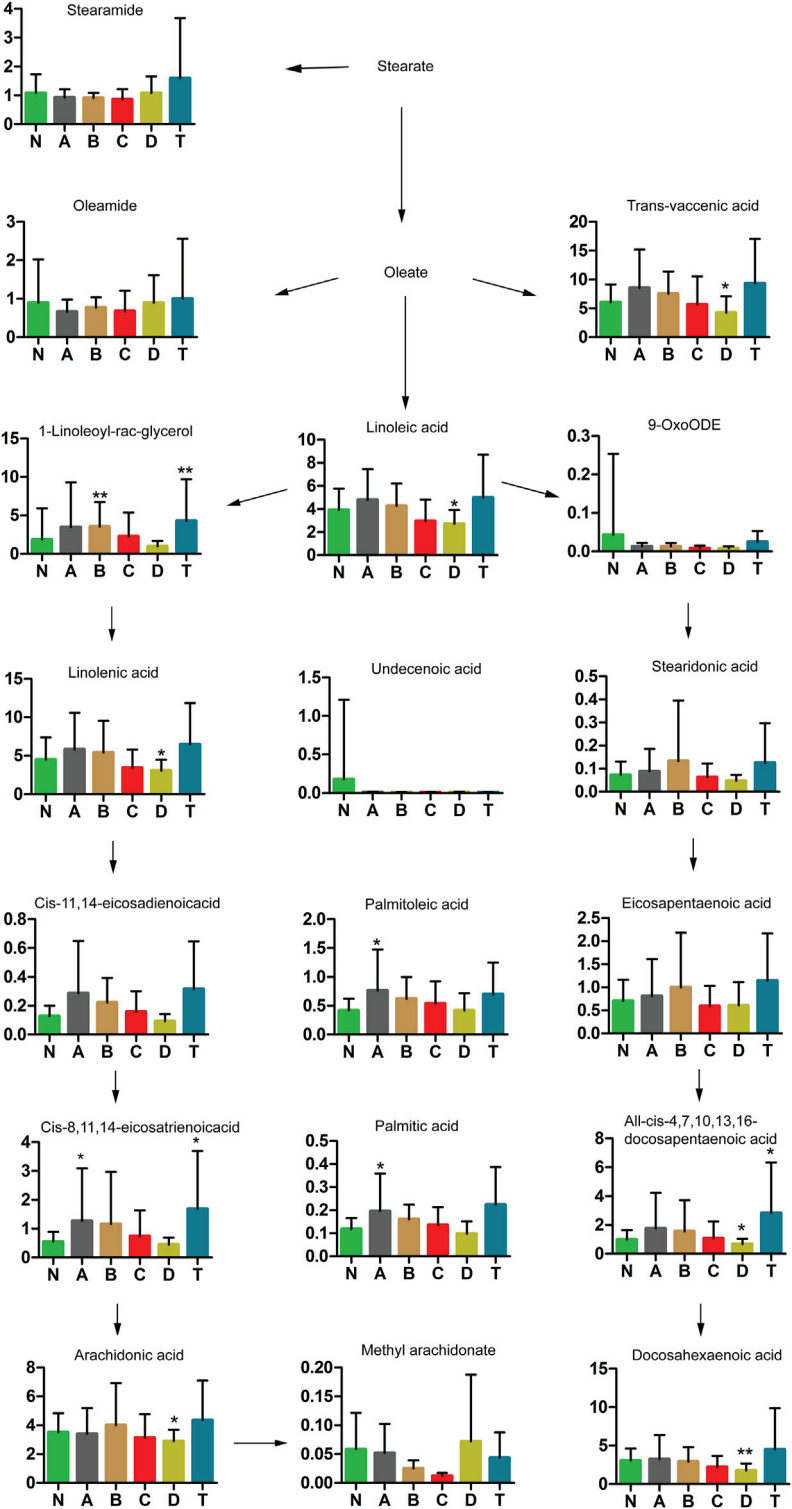
Changes in stearate metabolic pathway analysis of cerebral infarction. N, healthy controls, *n* = 41; A, attacks within 3 days of cerebral infarction, *n* = 31; B, onsets after 3 days but within 5 days of cerebral infarction, *n* = 17; C, onsets after 3 days but within 5 days of cerebral infarction, *n* = 13; D, attacks after 7 days of cerebral infarction, *n* = 19; T, cerebral infarction patients with glycosuria group, *n* = 20. All data are the mean ±SEM. *p < 0.05 vs. N group; **p < 0.01 vs. N group, two-tailed Mann–Whitney *U* test.

Our study further revealed that stearate metabolites changed in patients over different periods of cerebral infarction. As shown in Figure 6, trans-vaccenic acid, linolenic acid, linoleic acid, all-cis-4,7,10,13,16-docosapentaenoic acid, arachidonic acid, and docosahexaenoic acid (DHA) decreased after 7 days of cerebral infarction (D group). In addition, palmitoleic acid, cis-8,11,14-eicosatrienoic acid, and palmitic acid were increased within 3 days of cerebral infarction (group A) but gradually returned to normal levels. In particular, 1-linoleoyl-rac-glycerol was only elevated after 3 days and within 5 days of cerebral infarction (B group). Interestingly, three metabolites were significantly increased in the T group compared with those in the N group: 1-linoleoyl-rac-glycerol, cis-8,11,14-eicosatrienoic acid, and all-cis-4,7,10,13,16-docosapentaenoic acid. The aforementioned results showed that stearate metabolism was closely related to cerebral infarction during different periods.

### Relevant Changes of Monoacylglycerol and Sterols in Various Phases of CI Patients

**FIGURE 7:**
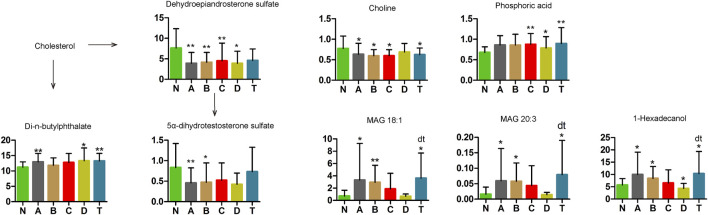
Changes in cholesterol metabolic pathway of cerebral infarction. N, healthy controls, *n* = 41; A, attacks within 3 days of cerebral infarction, *n* = 31; B, onsets after 3 days but within 5 days of cerebral infarction, *n* = 17; C, onsets after 3 days but within 5 days of cerebral infarction, *n* = 13; D, attacks after 7 days of cerebral infarction, *n* = 19; T, cerebral infarction patients with glycosuria group, *n* = 20. All data are the mean ±SEM. *p < 0.05 vs. N group; **p < 0.01 vs. N group; dt, *p* < 0.05 D group vs. T group; two-tailed Mann–Whitney *U* test.

Our results demonstrated that concentrations of three metabolites, monoacylglycerol (MAG) 18:1, MAG 20:3, and phosphoric acid, increased remarkably in groups A and B, but gradually returned to normal in group C (Figure 7). However, choline decreased noticeably from group A to group C, but gradually returned to normal in group D. In addition, 1-hexadecanol in A, B, and D was significantly lower, but higher in the T group, than in the normal group.

Dehydroepiandrosterone sulfate (DHEA), a cytoplasmic steroid, was significantly decreased in all CI groups compared to the N group. Moreover, 5α-dihydrotestosterone sulfate was significantly lower in the A and B groups than in N. In contrast, di-n-butylphthalate was elevated in the A, D, and T groups compared with the N group. These changes suggest that sterol metabolism is involved in the occurrence and development of CI.

### Critical Changes in LPC, PC, and FFA Levels in Different Periods of CI

**FIGURE8:**
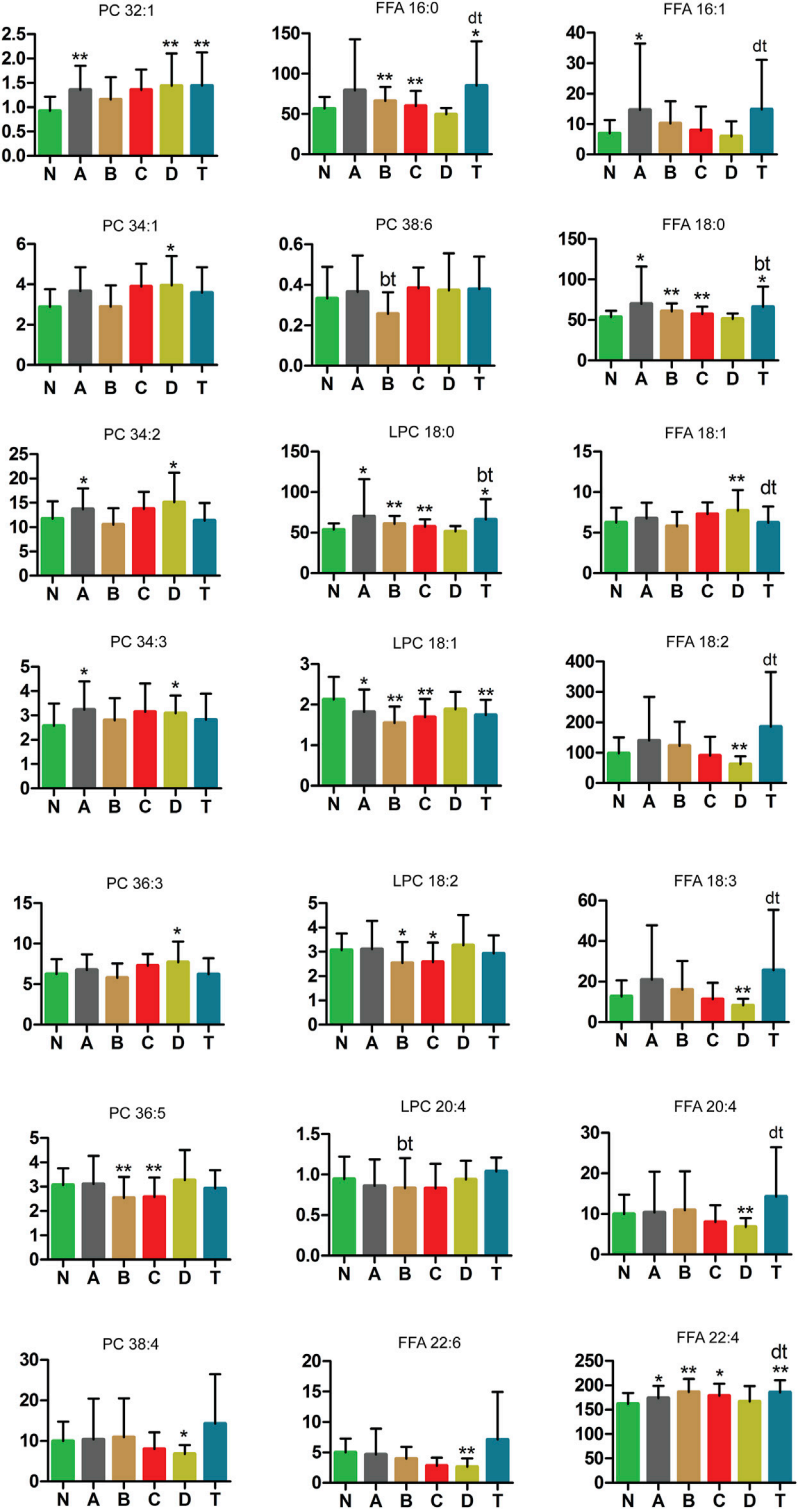
Changes in PC, LPC, and FFA metabolic pathway of cerebral infarction. N, healthy controls, *n* = 41; A, attacks within 3 days of cerebral infarction, *n* = 31; B, onsets after 3 days but within 5 days of cerebral infarction, *n* = 17; C, onsets after 3 days but within 5 days of cerebral infarction, *n* = 13; D, attacks after 7 days of cerebral infarction, *n* = 19; T, cerebral infarction patients with glycosuria, *n* = 20. All data are the mean ±SEM. *p < 0.05 vs. N group; **p < 0.01 vs. N group; bt, *p* < 0.05 B group vs. T group; dt, *p* < 0.05 D group vs. T group; two-tailed Mann–Whitney *U* test.

Lipids, or fats, are incredibly important to human health, and are one of the most difficult biomolecules to study. Body fat deposition and excessive FFA metabolism can lead to adverse health effects, including hyperlipidemia and obesity (Ebbert and Jensen, 2013). Four LPCs, eight PCs, and nine FFAs were measured in our study (Figure 8). LPC 18:1, LPC 18:2, FFA 18:2, FFA 18:3, FFA 20:4, FFA 22:6, PC 36:5, and PC 38:4 were lower in one or more CI groups than those in the N group. These results demonstrate three things—first, LPC 18:0 and LPC 18:1 had significant difference in the A, B, C and T groups compared with the normal group; LPC 18:0 was increased and LPC 18:1was decreased. Second, FFA 18:1, FFA 18:2, FFA 18:3, FFA 20:4, FFA 22:6, PC 34:1, PC 36:3, and PC 38:4 were markedly different between the D and N groups. Third, PC 36:5 and LPC 18:2 were all decreased in the B and C groups compared to the N group. In contrast, PC 32:1, PC 34:2, PC 34:3, FFA 16:1, FFA 18:0, and FFA 22:4 were higher in the A and/or D group than in the N group. Furthermore, FFA 18:0 and FFA 22:4 in the A, B, C, and T groups were significantly higher than those in the normal group. This is an important finding to understand lipid metabolism at various periods of the pathological process in cerebral infarction.

### Blood Biochemistry Tests in Various Periods of Cerebral Infarction and the Second Batch of Blood Samples Confirmation

**FIGURE 9:**
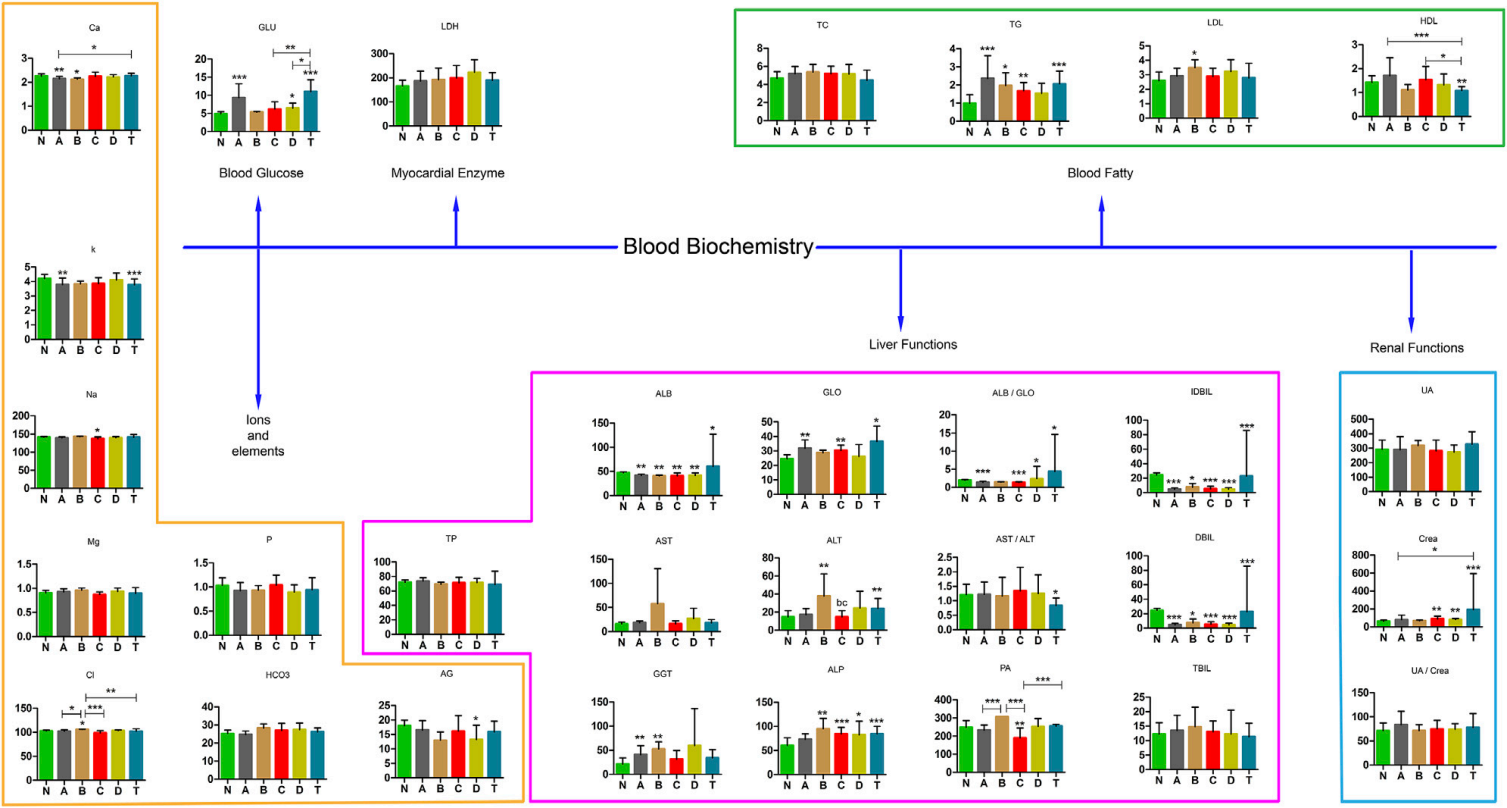
Blood biochemistry examination in various stages of cerebral infarction. N, healthy controls, *n* = 59; A, attacks within 3 days of cerebral infarction, *n* = 38; B, onsets after 3 days but within 5 days of cerebral infarction, *n* = 25; C, onsets after 3 days but within 5 days of cerebral infarction, *n* = 18; D, attacks after 7 days of cerebral infarction, *n* = 30; T, cerebral infarction patients with glycosuria, *n* = 32. All data are the mean ±SEM. *p < 0.05 vs. group; **p < 0.01 vs. group; ***p < 0.01 vs. group; two-tailed Mann–Whitney *U* test.

To better understand changes in substances in different periods of cerebral infarction, we analyzed the biochemical results of all the samples. Biochemical tests were classified into six categories: ions and elements, blood glucose, myocardial enzymes, liver function, blood fatty acids, and renal function. The detailed results are shown in Figure 9. Ca, K, Cl, TG, high density lipoprotein (HDL), albumin (ALB), globulin (GLO), ALB/GLO, indirect bilirubin (IDBIL), direct bilirubin (DBIL), alanine aminotransferase (ALT), glutamete transpeptidase (GGT), alkaline phosphatase (ALP), polymerase acidic protein (PA), and creatinine (Crea) were found to be significant in distinguishing cerebral infarction in different periods.

The test of the second batch of blood samples confirmed that the significant changes in 13 metabolites were consistent with the first batch. They were 5α-dihydrotestosterone sulfate, choline, di-n-butylphthalate, FFA 16:0, FFA 18:0, FFA 18:1, FFA 18:2, FFA 20:4, L-phenylalanine, PC 16:0–18:2 (PC 34:2), PC 34:3, phenylacetyl-L-glutamine, and phosphoric acid. The specific information is shown in Tables 1, 2 and Supplementary Table S1.

**TABLE 2:** Two batches results. Bold represents Figure: metabolites first appear in which figure.

Metabolite	Figure	Group	N vs. A	N vs. B	N vs. C	N vs. D	N vs. T	A vs. T	B vs. T	C vs. T	D vs. T
1-Heptanesulfonic acid	**7**	n first	**	*	*	*	**				**
		n second	***	**			***				
5α-Dihydrotestosterone sulfate	**7**	n first	**	*							
		n second		*							
Choline	**7**	n first	*	*	*		*				
		n second	**	***	***	**					
Di-n-butylphthalate	**7**	n first	**			*	**				
		n second		**	*	***					
FFA 16 : 0	**8**	n first		**	**		*				*
		n second		*	*		**				
FFA 18 : 0	**8**	n first	*	**	**		*		*		
		n second		*			*				
FFA 18 : 1	**8**	n first				**					*
		n second									*
FFA 18 : 2	**8**	n first				**					*
		n second									*
FFA 20 : 4	**8**	n first				**					*
		n second						*			*
L-Phenylalanine	**3**	n first	**	*	**	*	**				
		n second	*	**			*				
PC 16 : 00-18 : 2 (PC 34 : 2)	**8**	n first	*			*					
		n second	**	*		**	**				
PC 34 : 3	**8**	n first	*			*					
		n second				*					
Phenylacetyl-L-glutamine	**3**	n first	**	*		**					
		n second	*	*	*	*	*				
Phosphoric acid	**7**	n first			***	*	**				
		n second			***	*	**				

Note: N, healthy controls; n first=41, n second=18; A, time within 3 days of cerebral infarction, n first=31, n second=7; B, time after 3 days but within 5 days of cerebral infarction, n first=17, n second=8; C, time after 3 days but within 5 days of cerebral infarction, n first=13, n second=5; D, time after 7 days of cerebral infarction, n first=19, n second=11; T, cerebral infarction patients with glycosuria, n first=20, n second=12. *p < 0.05; **p < 0.01; ***p < 0.001; two-tailed Mann–Whitney *U* test.

## Discussion

CI is associated with high rates of disability and mortality. Changes in key metabolites over time in CI are unclear but need to be taken into account (Wang et al., 2018). It is vital that early diagnosis and key metabolite identification at various periods of CI are available for rational treatment and understanding the mechanism of the disease. The purpose of our study was to investigate key metabolites between different periods of CI and healthy people during the development of CI. This has not been investigated before.

### Metabolic Pathway Analysis

We identified the top ten pathways that differed significantly between the A, B, C, D, and T groups and N. (Figure 2). Phenylalanine (Phe) metabolism was involved in the development of cerebral infarction within 3 to 7 days. Phe, along with other physiologically active aromatic amino acids, is one of the essential amino acids that cannot be synthesized by humans and animals. Recently, it was shown that phenylalanine derivatives had protective effects on blood vessels due to their fibrinolytic effects (Koizumi et al., 2016). It is well known that blood clots in vessels can cause severe ischemic diseases such as cerebral infarction and myocardial infarction (Irie et al., 2014).The lower concentrations of Phe-Phe were found in groups A and B (Figure 3). Large amounts of Phe-Phe were consumed or their source reduction in the early periods of cerebral infarction merited further research.

There were three pathways that were differentially active only between the diabetic (T) and N groups: galactose metabolism, inositol phosphate metabolism, and ascorbate and aldarate metabolism. Our data are in good agreement with the data previously reported in literature. One study reported that increased atherosclerotic volume and plaques were correlated with IgE sensitization to α-galactose (Wu et al., 2017). Galactose, which increases satiety and mobilizes fat, can protect against type 2 diabetes (Charrière et al., 2017). It has been reported that inositol phosphate accumulation can cause accelerated platelet functions in diabetes mellitus (Ishii et al., 1991), and ascorbic acid (ascorbate) could reverse high glucose-induced increases in endothelial barrier permeability (Meredith et al., 2014).

### Analysis of Different Kinds of Metabolites

#### L-Isoleucine, GCDCA, and Glycocholate

The anti-inflammatory effect of indole derivatives (Figure 3) was shown in macrophages because they could inhibit phosphorylation of MAPK p38 and hinder DNA binding of transcription factor AP-1 (Finkin-Groner et al., 2017). L-isoleucine (Figure 3) has been found to promote the growth of bladder urothelial tumors by triggering the expression of amino acid transporters and tumorigenesis-related genes (Xie et al., 2013). Reports have also suggested that L-isoleucine is a potential biomarker to distinguish acute gout from asymptomatic hyperuricemia in patients with similar blood levels of uric acid (Luo et al., 2018).

As shown in Figure 4, GCDCA and glycocholate were markedly increased in the D group of cerebral infarction. GCDCA and glycocholate are conjugated bile acids which have higher water solubility and can increase fat emulsification and absorption (Hofmann and Mysels, 1992). Therefore, the increase in these two conjugated bile acids may be associated with the absorption of fats in patients with cerebral infarction.

#### Carnitines

The surprising fact is that medium-chain carnitine levels in the serum of the CI groups were lower than in healthy controls (Figure 5). In contrast, long-chain carnitines were higher and short-chain acylcarnitines showed no change in serum. This is the first time that this result was found in the serum of different periods of the CI patients. L-carnitine is an essential nutrient for transporting fatty acids that are needed to produce energy (da Silva Guimarães et al., 2017). L-carnitine also strengthens the effect of insulin on glycogen storage (Adeva-Andany et al., 2017). Convincing evidence from preclinical studies suggested that L-carnitine and its acetylation derivative, such as acetyl-L-carnitine, can improve the energy state of a pediatric brain injury model, reduce oxidative stress, and prevent subsequent cell death (Bak et al., 2016; Ferreira and McKenna, 2017), which are all important for minimizing ischemic effects. Studies have revealed that acetyl-L-carnitine plays a protective role against injury after ischemia in neurons *in vivo* (Zhang et al., 2012b). Our results suggest that medium-chain and long-chain acylcarnitines are significantly involved in the pathology of cerebral infarction.

#### Trans Fatty Acids

Moreover, it is unclear which of the trans fatty acids are important in different periods of cerebral infarction. We also looked at metabolites of fatty acid synthesis (Figure 6). Levels of trans-vaccenic acid, linoleic acid, linolenic acid, all-cis-4,7,10,13,16-docosapentaenoic acid, free arachidonic acid (ARA), and docosahexaenoic acid (DHA) were lower in group D, probably because of changes in anti-inflammation processes. Trans fatty acids (rTFA) can decrease inflammatory factors and increase oxidative stress factors in endothelial cells (Da Silva et al., 2017). Trans-vaccenic acid, an rTFA, can be desaturated to conjugate with linoleic acid (Santora et al., 2000), and linolenic acid can in turn attenuate inflammatory responses (Sergeant et al., 2016). Many derivates of docosahexaenoic acid, another fatty acid, had anti-inflammatory properties as well, even though mechanisms of their anti-inflammatory action have not been fully elucidated (Weylandt, 2016). ARA modulated the function of ion channels and several receptors and enzymes via activation as well as inhibition. Futhermore, metabolites derived from ARA oxidation did not initiate inflammation but contributed to it and, most importantly, led to the generation of mediators responsible for resolving inflammation and wound healing (Tallima and El Ridi, 2018). In addition, DHA exerted neuroprotective influence through activating the nuclear factor erythroid 2-related factor 2-antioxidant response element (Nrf2-ARE) pathway (Zhu et al., 2018). Exogenous DHA administration could protect neurons against Aβ1-42 oligomer-induced injury both *in vitro* and *in vivo*, partly by alleviating endoplasmic reticulum (ER) stress and preventing cell apoptosis (Champeil-Potokar et al., 2016).

We also found that palmitoleic acid, cis-8,11,14-eicosatrienoic acid, and palmitic acid were elevated within 3 days of cerebral infarction (A group) but returned to normal levels gradually in Figure 6. Palmitoleic acid (16:1, n-7), a monounsaturated fatty acid, is regarded as a lipokine. Studies have suggested it can increase fatty acid oxidation in the liver, improve blood fat, and alter macrophage differentiation (Maedler et al., 2003; Mozaffarian et al., 2010; Guo et al., 2012; Çimen et al., 2016). Palmitoleic acid is elevated in obese and metabolic syndrome patients (de Souza et al., 2018). These results reflected the activity of stearoyl coenzyme A desaturase-1, which could synthesize palmitate. In addition, palmitate has been shown to be elevated in liver and adipose tissues of obese patients (de Souza et al., 2018). Therefore, palmitoleic acid may play an anti-inflammatory role in the early period of cerebral infarction. Moreover, a biosynthetic pathway generating cis-8,11,14-eicosatrienoic acid (ETA) from arachidonic acid is also maybe an inflammatory factor. Reports revealed 5,8,11-eicosatrienoic acid had an inhibitory effect on angiogenesis (Hamazaki et al., 2012). Interestingly, an increase in similar derivatives of ETA will be found in skin aging and human liver cancer (Okazaki and Araki, 1978; Kim et al., 2010). A report found that non-esterified fatty acids accumulated when arteries were blocked for a long time. Palmitoleic acid and palmitic acid are all non-esterified fatty acids. Therefore, ETA and palmitic acid are probably inflammatory factors in the early periods of cerebral infarction.

We also detected an increase in 1-linoleoyl-rac-glycerol in the B and T groups. Previously reported 1-palmitoyl-2-linoleoyl-3-acetyl-rac-glycerol has an immunomodulatory function (Hwang et al., 2015) and modulates eosinophil chemotaxis by regulating CCL26 expression in epithelial cells (Jeong et al., 2016). Whether 1-linoleoyl-rac-glycerol has similar effect in CI needs further study.

#### MAG 18:1, MAG 20:3, Choline, Phosphoric Acid, 1-Hexadecanol, DHEA, DHEA Sulfate, and Di-N-butylphthalate

In Figure 7, our study found that concentrations of three metabolites, MAG 18:1, MAG 20:3, and choline, were remarkably changed in the A and B groups but returned to normal gradually by C or D periods. Glucose can specifically activate triacylglycerol (TAG) to produce diacylglycerol (DAG), which indirectly produces MAG 18:1 (Pearson et al., 2016). Pearson et al. also confirmed that MAG 18:1, PC, and DAG/SM were closely linked to metabolic stimulation-secretory coupling (Pearson et al., 2016). However, the mechanism of these two MAGs needs further exploration. Choline, a precursor of acetylcholine, can protect the heart from damage by inhibiting apoptosis of ischemic cardiomyocytes and excessive autophagy (Hang et al., 2018). Choline precursors can promote the repair and growth of cell membranes in neurological diseases (Delaunay et al., 2017). Phosphoric acid (H_3_PO_4_), be detected an increase in groups C, D and T, has the highest intrinsic proton conductivity of any known substance and is frequently an inorganic acid in acylation and phosphocreatine (Vilčiauskas et al., 2012).

In groups A, B, and T, 1-hexadecanol was significantly higher than in group N, but lower in the D group (Figure 7). In many cell functions, 1-hexadecanol competitively displaces cholesterol from phospholipids (Ratajczak et al., 2007). Overexpressed acetyl-CoA carboxylase gene and knocking out the negative regulator of the inositol-1-P synthase gene in phospholipid metabolism improved 1-hexadecanol production (Feng et al., 2015). Therefore, 1-hexadecanol may be involved in the physiological processes involving cholesterol and steroidogenesis relating to CI. In humans, the circulating concentrations of DHEA and DHEA sulfate (DHEAS) decrease markedly during aging and have been implicated in age-associated cognitive decline (Sorwell and Urbanski, 2010). Plasma DHEA can inhibit atherosclerotic intimal hyperplasia (Herrington et al., 1990). Di-N-butylphthalate (DBP), a ubiquitous environmental pollutant used for plastic coating and in the cosmetics industry, has toxic effects on body health (Jiang et al., 2017; Kong et al., 2018). Decreased DHEA and DHEA levels and increased DBP might be associated with cognitive decline and accumulation of toxicity in older patients. This is precisely why being in the older age group has higher odds of CI as compared with the younger age group.

#### LPC, PC, and FFA

As shown in Figure 8, this study also demonstrated the following significant findings in LPC, PC, and FFA. LPC 18:0 and LPC 18:1 were decreased in the A, B, C, D, and T groups compared with the healthy control group. Instead, LPC 18:2 was decreased in the B and C groups compared to the N group. This also shows that LPC 18:0 and LPC 18:1 are involved in each periods of CI but LPC 18:2 plays a prominent role in the intermediate period. LPCs have various stimulated effects on many types of immune cells (Takatera et al., 2007). Endothelial lipase (EL) and EL-generated LPCs promoted IL-8 expression in endothelial cells (Croner et al., 2012; Essaji et al., 2013; Pearson et al., 2016). LPC species were capable of eliciting production of PGI(2) (Riederer et al., 2010). Low plasma LPC 18:2 had previously been shown to predict impaired glucose tolerance, insulin resistance, coronary artery disease, and memory impairment (Gonzalez-Freire et al., 2018). Expression of recombinant NTE (rNTE) in Neuro-2a cells altered their phospholipid balance by lowering LPC-16:0 and LPC-18:0 and by elevating glycerophosphocholine (Vose et al., 2008a). During this process, the cytotoxicity of phosphatidylcholine, LPC 16:0/18:1, or 16:0/18:2 was not altered (Kishimoto et al., 2002; Vose et al., 2008b). While LPC 18:1 induced a weak and transient increase in cyclooxygenase (COX)-2 mRNA but not protein in vascular endothelial cells, LPC 18:2 increased COX-2 protein, without impacting mRNA (Brkić et al., 2012). Since LPCs are endogenous ligands for the GPR119 receptor, LPCs could mediate glucose-stimulated insulin secretion (Drzazga et al., 2018).

In addition, PC 32:1, PC 34:1, PC 34:2, PC 34:3, and PC 36:3 were higher in the A and/or B group than in the N group (Figure 7). PC 36:5 was decreased in the B and C groups compared to the N group and PC 38:4 was markedly decreased in the D group. These also suggest that PCs are obvious distinctions in different periods of CI. PCs are major components of the phospholipids in vascular endothelial cells. Polyene phosphatidylcholine has an anti-inflammatory effect (Pan et al., 2017). Anti-PC antibodies might be risk factors with cerebral infarction (Korematsu et al., 2017). PC could reduce the oxidative stress in the sciatic nerve and had protective effects against peripheral neurotoxicity (Kim et al., 2018). PC caused lipolysis and apoptosis in adiposity through the tumor necrosis factor alpha-dependent pathway (Jung et al., 2018). PC containing unsaturated long-chain acyl groups also prevented neuronal death caused by amyloid β-protein (Aβ) 1–42 (Ko et al., 2016).

Moreover, in Figure 8, FFA 16:0, FFA 16:1, FFA 18:0, and FFA 22:4 were higher in the A and/or B group than in the N group. In contrast, FFA 18:1, FFA 18:2, FFA 18:3, FFA 20:4, and FFA 22:6 were markedly different between the D and N groups. Furthermore, FFA 18:0 and FFA 22:4 in the A, B, C, and T groups were significantly higher than those in the healthy control group. Taken together, these results suggest that FFAs show different changes in different periods of cerebral infarction. Both saturated and unsaturated FFAs play important roles in energy metabolism, biological mediators, and biological structures (Offermanns, 2014). They also had cellular effects by activating G protein-coupled receptors from FFA1 to FFA4. A previous study suggested that several FFA receptors could be used as therapeutic targets for type 2 diabetes and inflammation (Offermanns, 2014). FFA was dramatically and acutely changed under ischemic stroke in a mouse model (Golovko and Golovko, 2018). Concentrations of elevated FFA in plasma were significantly correlated with ischemic lesion volume and incidence in non-arterial embolic stroke patients, but not with high-risk cardiac embolism sources (Chung et al., 2017). A study demonstrated that elevated serum and cerebrospinal fluid FFA levels were associated with unfavorable functional outcomes in subjects with acute ischemic stroke (Duan et al., 2017). Evidence has shown that the effect of insulin on the release of FFA and leptin is adipose deposit–dependent (Wueest and Konrad, 2018). High FFA levels are a common feature of obesity and are the primary cause of endothelial dysfunction (Han et al., 2015). FFA not only mediates adverse metabolic effects, such as reducing carbohydrate metabolism, but also provides a causal relationship between obesity and the pathological development of type 2 diabetes (Spiller et al., 2018). As discussed previously, our results revealed that key metabolic pathways and risk metabolites play vital roles in the different periods of cerebral infarction.

Although clinical biochemical indices have suggested some information and instruments can help us judge the timing and development stages of cerebral infarction, we understand very little about key metabolic pathways and risk metabolites pose in different periods of CI. Risk metabolites showed significant differences in cerebral infarction in different periods, which were complementary to each other and provided the basis for us to better understand the pathology of CI.

There are some limitations of the study that are important to discuss. Even though there were relatively equal numbers of gender and age in each cerebral infarction group, we were underpowered to evaluate associated diseases (except for blood sugar), dietary supplements, and diet differences. Furthermore, the second batch samples used for validation was relatively modest (*n* = 18, Healthy controls, 43 for validation), although 13 prediction metabolites were partly validated (Supplementary Table S2 illustrates the results of the validation relative to the first batch). However, it will still need to be replicated in an independent manner and with enough quantity of samples in the future. Moreover, because most cerebral infarction recurrences could only be judged by instruments (CT or NMR), we noted that our study was intended to be an illustration of complimentary methods that might improve clinical prediction of patients at different stages of cerebral infarction.

## Conclusion

**FIGURE 10:**
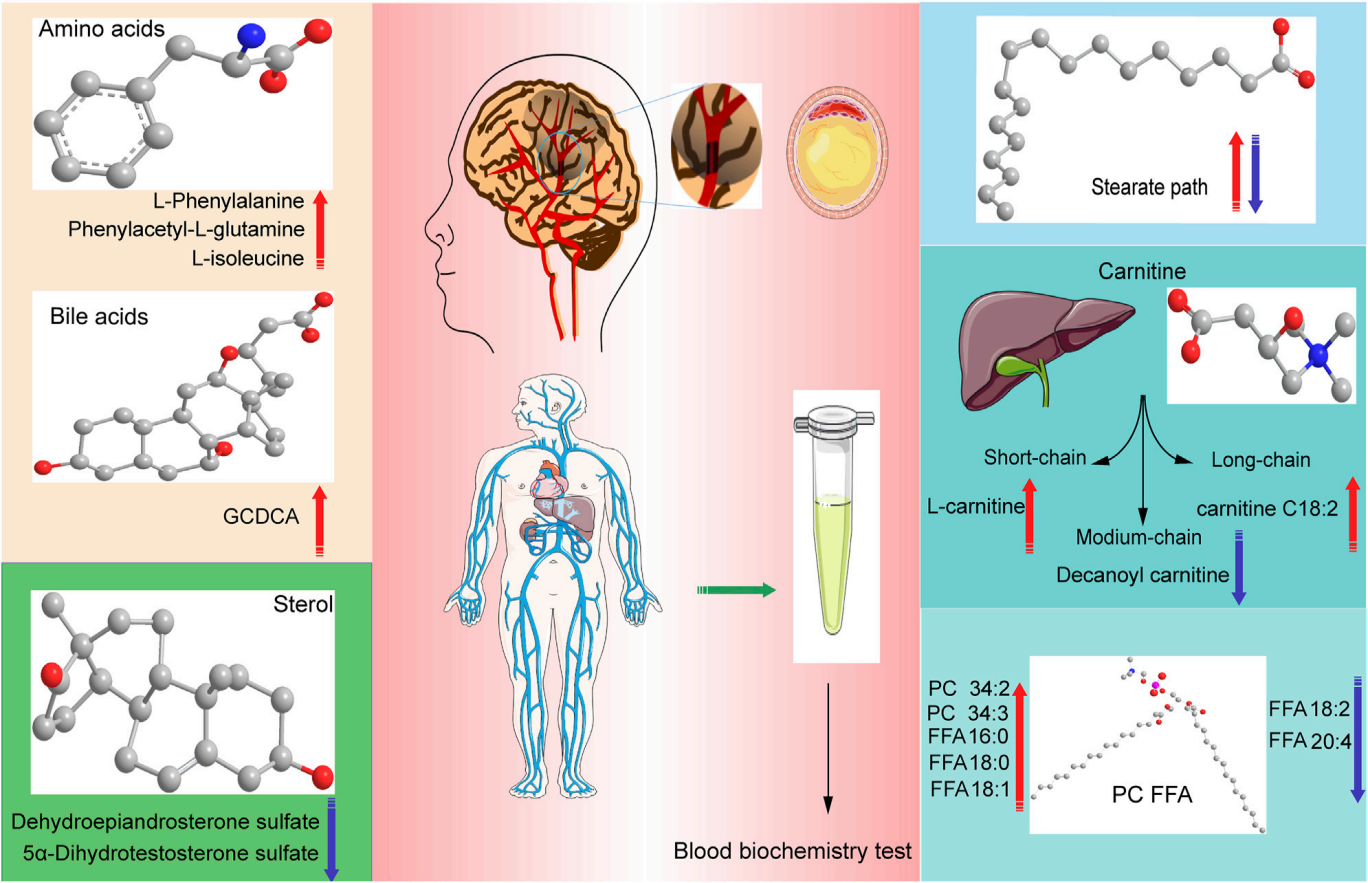
Graphical abstract, conclusions of our study in different periods of Cerebral Infarction in Human. The red arrows indicate that it is elevated in this kind of metabolites, the blue arrows indicate that it is elevated in this kind of metabolites suffering from cerebral infarction.

We found some key metabolic pathways between CI groups of different periods and healthy controls Figure 10. For example, valine, leucine, and isoleucine biosynthesis; valine, leucine, and isoleucine degradation; and tryptophan metabolism, ubiquinone, and other terpenoid–quinone biosynthesis differed significantly between the B (intermediate stage) and N groups. Specific data are detailed in the *Metabolic pathway analysis in different periods of CI by the online MetaboAnalyst website section*.

We also revealed phenylalanine metabolism, medium-chain acylcarnitines, long-chain acylcarnitines, choline, DHEA, LPC 18:0, LPC 18:1, FFA 18:0, FFA 22:4, TG, ALB, IDBIL, and DBIL played vital roles in the development of different periods of CI from Figure 3 to Figure 9. Interestingly, Phe-Phe, carnitine C18:1, palmitic acid, cis-8,11,14-eicosatrienoic acid, palmitoleic acid, 1-linoleoyl-rac-glycerol, MAG 18:1, MAG 20:3, phosphoric acid, 5α-dihydrotestosterone, Ca, K, and GGT were the major components in the early period of CI. GCDCA, glycocholate, PC 36:5, LPC 18:2, and PA showed obvious changes in the intermediate time. In contrast, trans-vaccenic acid, linolenic acid, linoleic acid, all-cis-4,7,10,13,16-docosapentaenoic acid, arachidonic acid, DHA, FFA 18:1, FFA 18:2, FFA 18:3, FFA 20:4, FFA 22:6, PC 34:1, PC 36:3, PC 38:4, ALP, and Crea displayed changes in the later time. It is very different from the other that PC 32:1, PC 34:2, PC 34:3, FFA 16:1, FFA 18:0, and FFA 22:4 were higher in the A and/or D groups than in the N group. The specific data are detailed in Supplementary Table S3.

Furthermore, three pathways that differed only between the T (CI patients complicated with high blood glucose) and N groups were found (Supplementary Table S3). They were galactose metabolism, inositol phosphate metabolism, and ascorbate and aldarate metabolism. Futhermore, many critical metabolites incluing GCDCA, 1-linoleoyl-rac-glycerol, cis-8,11,14-eicosatrienoic acid, all-cis-4,7,10,13,16-docosapentaenoic acid, di-n-butylphthalate, MAG 18:1, MAG 20:3, phosphoric acid, choline, LPC 18:0, LPC 18:1, PC 32:1, FFA 16:0, FFA 18:0, FFA 22:4, K, TG, HDL, ALB, GLO, ALB/GLO, ALT, ALP, IDBIL, DBIL, and Crea were significantly different in the T group compared with those in the N group.

The number of critical metabolites underlying cerebral infarction is increasing, and in combination with the advancement in studying biological mechanisms correlated with cerebral infarction, the field may gradually move toward greater precision in diagnosis, clarifying the pathological mechanisms and improving outcomes through personalized treatment.

## Data Availability

The original contributions presented in the study are included in the article/Supplementary Materials; further inquiries can be directed to the corresponding authors.

## Glossary

ALP: alkaline phosphatase
DHA: docosahexaenoic acid
LC: l-carnitine
LPC: lysophosphatidyl cholines
MAG: monoacylglycerol
OPLS-DA: orthogonal partial least-squares discriminant analysis
PE: phosphatidylethanolamine
TAG: triacylglycerol
ALC: acetyl-l-carnitine
ALT: alanine aminotransferase
ALB: albumin
AA: amino acids
BA: bile acids
CI: cerebral infarction
CoA: coenzyme A
Crea: creatinine
COX: cyclooxygenase
DHEA: Dehydroepiandrosterone sulfate
DHEAS: DHEA sulfate
DAG: diacylglycerol
DBP: Di-N-butylphthalate
DBIL: direct bilirubin
ER: endoplasmic reticulum
EL: endothelial lipase
ARA: free arachidonic acid
FFA: free fatty acids
GLO: globulin
GGT: glutamete transpeptidase
GCDCA: glycochenodeoxycholate
HDL: high density lipoprotein
IDBIL: indirect bilirubin
Nrf2-ARE: nuclear factor erythroid 2-related factor 2-antioxidant response element
PC: phosphatidylcholines
PA: polymerase acidic protein
PCA: principal component analysis
QC: Quality control
rNTE: recombinant NTE
SM: sphingomyelin
rTFA: Trans fatty acids
TG: triglyceride
UPLC-Triple-TOF-MS: ultra performance liquid chromatography-triple time of flight mass spectrometry
